# Chemical accuracy for ligand-receptor binding Gibbs energies through multi-level SQM/QM calculations†

**DOI:** 10.1039/d4cp01529k

**Published:** 2024-07-19

**Authors:** Froze Jameel, Matthias Stein

**Affiliations:** a Molecular Simulations and Design Group, Max Planck Institute for Dynamics of Complex Technical Systems Sandtorstrasse 1 39106 Magdeburg Germany matthias.stein@mpi-magdeburg.mpg.de

## Abstract

Calculating the Gibbs energies of binding of ligand-receptor systems with a thermochemical accuracy of ± 1 kcal mol^−1^ is a challenge to computational approaches. After exploration of the conformational space of the host, ligand and their resulting complexes upon coordination by semi-empirical GFN2 MD and *meta*-MD simulations, the systematic refinement through a multi-level improvement of binding modes in terms of electronic energies and solvation is able to give Gibbs energies of binding of drug molecules to CB[8] and β-CD macrocyclic receptors with such an accuracy. The accurate treatment of a small number of structures outperforms system-specific force-matching and alchemical transfer model approaches without an extensive sampling and integration.

## Abstract 2

The semi-empirical quantum chemical exploration of the conformational space of ligand-receptor complexes followed by systematic QM refinement of poses leads to Gibbs energies of binding with thermochemical accuracy.

## Graphical abstract

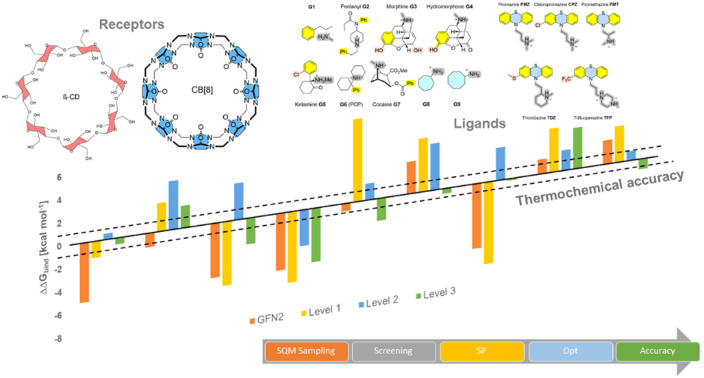

## Introduction

The calculation of ligand-receptor Gibbs energies of binding with thermochemical accuracy is still a major challenge for state-of-the art computational approaches.^1,2^ For example in drug discovery,^3^ an accuracy of, at most, 1–2 kcal mol^−1^ can be achieved from quantitative modelling using force fields and extensive sampling techniques to compute *relative* Gibbs energies of binding.^3^ SAMPL (Statistical Assessment of the Modeling of Proteins and Ligands) are blind challenges to validate and improve state-of-the-art computational methods as predictive tools in drug design. Summaries of recent SAMPL challenges can be found in ref. 4, 9 and 10. In particular, charged receptor entities represent a challenge in terms of accuracy.^4^ Here, we show that this thermochemical accuracy can also be obtained using a combination of a fast tight-binding quantum chemical exploitation of the conformational space, plus a systematic and sequential refinement of solvation and interaction energies.^5,6^ The semi-empirical quantum chemical (SQM) GFN2 Hamiltonian allows an efficient exploration of the conformational space of complex molecular systems without the need for a re-parametrization of interaction terms even for non-standard binding situation, such as open-shell transition metal complexes.^7,8^

For these blind predictions of ligand-receptor Gibbs energies of binding, macrocyclic containers such as cucurbit[*n*]urils (CB[*n*])^11,12^ and cyclodextrins (CDs)^13,14^ with unreleased experimental data are chosen.

**Fig. 1:**
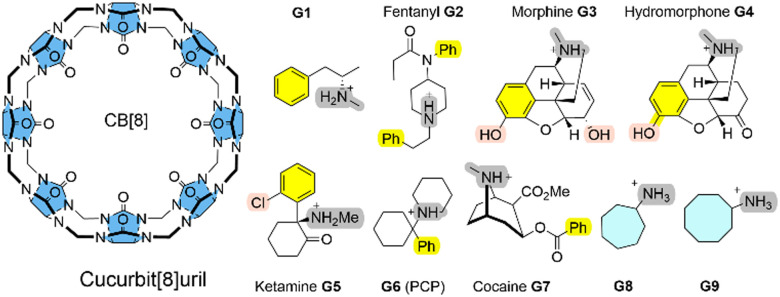
CB[8] host and guest structures G1–G9.

Here, we systematically refine the Gibbs energies of binding from conformers generated through SQM sampling for the ‘drugs of abuse’ molecules to the CB[8] receptor of the SAMPL8 host–guest challenge. This challenge focused on binding of the CB[8] host to opioid drug molecules including fentanyl (**G2**), morphine (**G3**), ketamine (**G5**), and cocaine (**G7**) (see Fig. 1).^9^ It also included previously considered cycloheptanamine and cyclooctanamine molecules (G8 and G9). Experimental data were obtained from isothermal titration (ITC) and NMR spectroscopy.^15^ This set of SQM conformer–rotamers was used to investigate whether the mean absolute deviation of 3.16 kcal mol^−1^ from GFN2-*x*TB^16^ could be reduced using the suggested systematic refinement of both electronic energies and solvent description.^5^

In addition, we here present calculated Gibbs energies of binding of phenothiazine drug molecules to the β-cyclodextrin receptor, which was part of SAMPL9 challenge.^10^ Cyclodextrins are versatile and flexible receptors that can incorporate various types of molecules and modify their molecular properties. They also have applications in drug delivery. The Gilson group provided experimental thermodynamic data for different cyclodextrins and ligand molecules which were only later released and published.^17^ The amphiphile receptor centre with a hydrophobic cavity plus hydrophilic surface-exposed hydroxyl groups in complex with large and flexible ligands poses a further level of complexity to calculate Gibbs energies of binding.

The conformer-rotamer ensembles (CRE) for SAMPL8 and SAMPL9 ligand complexes with cucurbit[8]uril (CB[8]) and β-cyclodextrin (β-CD) hosts, respectively, were generated upon manually positioning the ligand inside the receptor. Ensembles of non-covalent binding poses were generated using GFN2-*x*TB with an automatic exploration of the low-energy molecular chemical space CREST.^5,18^ The CENSO workflow^6^ including different levels of quantum chemical refinement plus thermostatistical corrections and solvent modelling was as used to systematically refine the calculated Gibbs energies of binding.

## Results and discussion

### Cucurbit[8]uril binding to ‘drugs of abuse’

Cucurbit[*n*]urils (CB[*n*]s) are a family of macrocyclic receptors with a number of *n* glycoluril units^11,19^ with tuneable radii and properties. Their molecular recognition of a broad range of guest molecules with high affinities^20^ has led to numerous applications, such as a building block in a supramolecular polymer networks with high stretchability^21^ or as a carrier for anti-cancer drugs.^22^ CB[8] is the largest member of this family with a cavity diameter of 8.8 Å and a cavity volume of 479 Å^3^.^23^ The SAMPL8 challenge focused on CB[8] receptor binding to ligands which are ‘drugs of abuse’, including morphine, hydromorphone, methamphetamine, cocaine, and others (see Fig. 1).^9^ Also cycloheptanamine and cyclooctanamine (G8 and G9) were included from previous SAMPL challenges. The thermodynamics of binding of CB[8] towards the drugs of abuse was determined by a combination of ^1^H NMR spectroscopy and isothermal titration calorimetry in phosphate buffered water. CB[8] and guest molecules form 1 : 1 complexes with experimental Gibbs energies of binding in a range between −7.05 (for G1) and −14.07 kcal mol^−1^ (for G6).^15^ High affinity measurements have attributed this to an interaction between the guest's ammonium group and the carbonyl oxygen of CB[8].

Curcurubituril[8] is a rather rigid host with only a few conformers accessible.^16^ However, with certain force fields, cucurbituril hosts have been observed to collapse during MD simulations. The sampling of conformers of CB[8] receptor and receptor–ligand complexes showed that this is not the case for GFN2-*x*TB.^16^ A conformational search using an implicit solvation model in absence of any constraints gave only one single CB[8] host structure. The ligand molecules of SAMPL8 are rather small in size with only few rotatable bonds (Fig. 1). This leads to only 4–8 unique structures in the conformer–rotamer ensemble (CRE) of CB[8] in complex with large and rigid morphine (G3) and hydromorphone (G4), but up to 137 unique complex structures with the rather flexible fentanyl molecule (G2). The subsequent energetic ranking must thus be able to accurately calculate small energy differences between a large number of binding modes in order to give reliable Gibbs energies of binding. The CREs from our SAMPL8 GFN2-*x*TB/MetaMD/GBSA work^16^ were refined using a systematic improvement of description of electronic energies and solvation. We have used a three level approach with increasing refinement thresholds to reduce the number of structures to be considered at the next level (see Computational details).

The incremental increase in accuracy of electronic structure method, description of effect of solvation (see Computational details) and refined energy thresholds to omit higher lying structures significantly reduces the number of structures retained at leach level (see Fig. S2, ESI†). For example, for CB[8]·G3 (morphine) and CB[8]·G4 (hydromorphone) only one structure after ‘level 2’ is sufficient to give reliable Gibbs energies of binding (see below). Also for the flexible fentanyl (G2), when in complex with CB[8], a mere two remaining structures after ‘level 2’ give excellent calculated Gibbs energies of binding (see below). The largest decrease of tentative complexation structures is always seen when the level of DFT treatment is going from the fast and approximate B97-D3 with a small basis set to the *meta*-GGA r^2^SCAN-3c (see Fig. S1, ESI†).

**Fig. 2:**
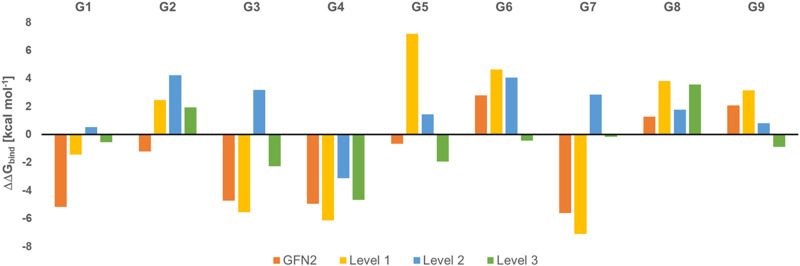
Deviation of calculated Gibbs energies of binding from experiment (ΔΔ*G*_bind_, in kcal mol^−1^) for G1 to G9 ligands to the CB[8] receptor.

Fig. 2 shows the deviation of calculated Gibbs energies of binding of the CB[8] receptor to ligands G1–G9 from experiment. The numbers reported are the Boltzmann-weighted averages of conformers at each level. The original GFN2 CREs had a mean absolute deviation (MAD) of 3.16 kcal mol^−1^ from experiment which is already close to the top-ranked force matching approach with a MAD of 2.03 kcal mol^−1^ in SAMPL8.^9^ Since calculations at ‘level 0’ are mere approximate single-point energies and not Gibbs energies of binding to remove high-lying complexes, they cannot be directly compared with experiment and are not discussed further (see Table S2 for numerical results, ESI†). A negative ΔΔ*G*_bind_ indicates an overbinding (too negative Gibbs energies) in Fig. 2. GFN2 systematically overestimates the Gibbs energies of binding (only for cyclic amines G8 and G9 an underestimation by 1–2 kcal mol^−1^ is seen). The composite *m*GGA method r^2^SCAN-3c at ‘level 1’ gives significantly better Gibbs energies of binding even for single-point calculations. This method was originally found to outperform hybrid functionals in terms of conformational energies at a significantly lower computational cost whereas non-covalent interactions are about as well described as with hybrid functionals.^24^ However, from Fig. 2 it becomes apparent that a structural re-optimization of GFN2 structures in combination with an improved description of solvation free energies (from ALPB to COSMO-RS) significantly reduces the deviation from experiment (MAD decreases from 4.6 kcal mol^−1^ at ‘level 1’ to 2.45 kcal mol^−1^ at ‘level 2’). This reduced MAD results in part from improved electronic energies (by 1.52 kcal mol^−1^) and solvation (by 0.64 kcal mol^−1^; see Table S2, ESI†). The hybrid *meta*-GGA PW6B95 at ‘level 3’ gives an additional reduction of MAD by ∼1 kcal mol^−1^ (see Table 1) and a final MSE of −0.6 kcal mol^−1^.

**Table 1:** Analysis of error of calculated Gibbs energies of binding in kcal mol^−1^ for SAMPL8 entries

	MSE^a^	SEM^b^	MAD^c^
GFN2	−1.79	1.13	3.16
Level 0	−16.84	1.71	16.84
Level 1	+0.11	1.78	4.61
Level 2	+1.75	0.76	2.45
Level 3	−0.60	0.79	1.82

a Mean signed error.

b Standard error of mean.

c Mean absolute deviation.

In the global statistical analysis of SAMPL8,^9^ there were 5 ligand-receptor CB[8] complexes which had a root mean square error of 4 kcal mol^−1^ or larger among all submissions. These were CB[8]·G1, CB[8]·G3, CB[8]·G4, CB[8]·G6, and CB[8]·G7 (see Fig. 1). The majority of methods tended to underestimate the Gibbs energies of binding for these systems, except for one with a Gibbs energy of binding ∼8 kcal mol^−1^ too large. Our level 2/level 3 results have a deviation from experiment of +0.5/−0.5 kcal mol^−1^ (G1); +3.2/−2.3 kcal mol^−1^ (G3); −3.2/−4.7 kcal mol^−1^ (G4); +4.1/−0.4 kcal mol^−1^ (G6); +2.8/−0.16 kcal mol^−1^ (for G7). This shows, that the CENSO refinement of a large number of GFN2 binding modes is competitive with and, in some instances, outperforming previous submissions to SAMPL8. Ligands G3 (morphine) and G4 (hydromorphone) appear to be intrinsically difficult to describe for any computational method. These are among the most complex ligand entities in SAMPL8 with multi-ring heterocycles, decorated by several functional groups and a protonated tertiary amines as part of a heterocycle. Due to their chemical environment these are notoriously difficult to describe for solvation models (see below). For ligand systems G1, G6, and G7 the extra computational cost to go from the *meta*-GGA type r^2^SCAN-3c (level 2) to the hybrid *meta* exchange-correlation functional PW6B95 (level 3) seems to be worth since the final MSE for these compounds is the best for those entries in SAMPL8.

For the CB[8] host, the overall top-performer in SAMPL8 so far have used classical bonding and non-bonding parameters obtained *via* QM force-matching (FM) methods, QM-derived atomic charges and fitted bonded parameters with a final MAD of 2.03 kcal mol^−1^.^9,25^ Derivation of QM charges and the molecular force matching was computationally expensive and required 10 000s of DFT force calculations for each complex. As an alternative approach, with much less extensive sampling and a systematic refinement of QM interaction energies, our results with a MSE of −0.60 kcal mol^−1^ and a MAD of 1.82 kcal mol^−1^ outperform the FM and MD ansatz. Such an accuracy is achievable even when considering two to three orders fewer structures, depending on the level of refinement (see Fig. S2, ESI†).

### Gibbs energies of binding of β-cyclodextrin to phenothiazine drugs

**Fig. 3:**
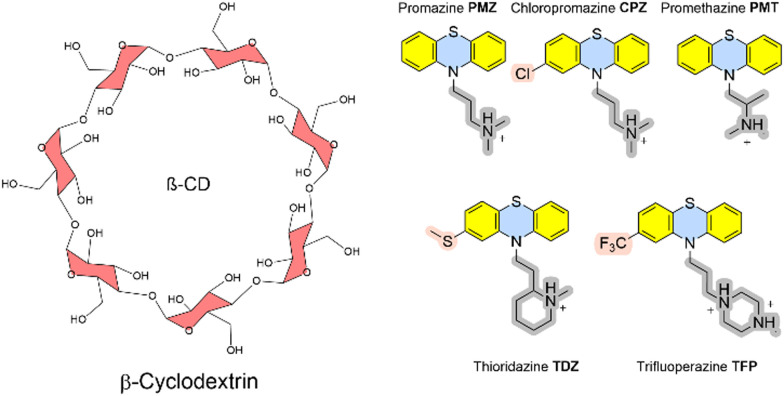
Structures of the β-cyclodextrin host and phenothiazine-derived guest molecules.

The recent SAMPL9 competition included the prediction of Gibbs energies of binding between β-cyclodextrin and five phenothiazine-based antipsychotic drugs (see Fig. 3).^26^ In β-cyclodextrin, seven glucose subunits are α-1,4 linked to give a cone-shaped host with ∼6 Å diameter, a hydrophobic interior and a slightly hydrophilic exterior surface. Cyclodextrin containers bind a range of guest molecules in aqueous solution by both hydrophobic and polar interactions and confer solubility, stability, and bioavailability to drug molecules and are thus used as drug carriers.^13,14^ Phenothiazines are a class of first-generation drugs for anti-psychotic medications, such as schizophrenia, bipolar disorders, and other psychotic disorders with delusional manifestation.^27^ They represent a class of nitrogen and sulfur-containing heterocyclic drug molecules. Some of the phenothiazines (CPZ, TDZ, TFP) are substituted at the phenothiazine entity to give an asymmetric guest molecule. Increasing hydrophobicity at position 4 (–Cl and –CF_3_ groups), varying alkyl chain lengths, branching and different terminal tertiary amines were found to be critical determinants for their biological activity.^28^

The Gilson group found that β-CD binds to phenothiazine drug entities with good affinity and extended the investigation to phenothiazine derivatives. They possess favourable solubilities, and constitute a new class of molecule family that was never explored in any SAMPL challenge in the past. Experimental Gibbs energies of binding were obtained from ITC and NMR to characterize the non-covalent interactions and revealed the formation of 1 : 1 complexes.^17^ Gibbs energies of binding are in a very narrow range between −4.5 and −5.7 kcal mol^−1^. Chemical modifications at the heterocyclic core and/or aliphatic chain lengths increase the degree of conformational flexibility of the ligand; in combination with the flexible receptor this set represents a further level of challenge to computational approaches.

Whereas the CB[8] receptor was a rigid macrocyclic entity, the GFN2 conformational search of β-CD gave 56 unique conformers in the CRE, albeit some were high in energy and removed in subsequent QM refinement iteration steps. This shows that the iterative refinement procedure with increasing thresholds is an efficient way to filter out many high-lying conformers and to retain also few unique structures in a narrow energy window (after ‘level 3’ only two conformers were below the Boltzmann threshold of 99%). The SAMPL8 ‘drugs of abuse’ guests were small molecules with only a small number of unique conformers apart from G2 (see above). The free phenothiazines give rise to a larger number of unique conformers after CREST searches. However, the sequential QM refinement reduces the number of entries in a certain energy window above the minimum significantly by a factor of 5–10 (see Fig. S3, ESI†). For the phenothiazine-β-CD complexes, the conformational search also yielded more unique structures compared to SAMPL8 but most of them were removed during sequential refinement of electronic energies and solvation modelling.

**Fig. 4:**
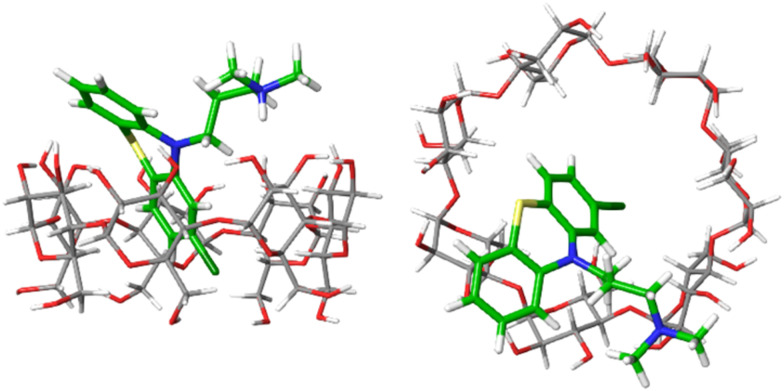
Top-ranked binding pose of CPZ binding to β-CD. Left: Side view, right: top view.

Fig. 4 shows one example of the top-ranked pose of CPZ guest in complex with β-CD receptor. In general, the binding modes of highly ranked phenothiazine poses are in agreement with the structural interpretation of NMR studies. The β-CD receptor has two binding faces: the ‘primary’ is made up by seven primary alcohols (bottom of receptor in Fig. 4(left)), and the ‘secondary’ is consisting of fourteen alcohol groups on the other side of the receptor (top of Fig. 4(left)). Part of the phenothiazine moiety is located at the secondary face of the host and part of the drug penetrates deep into the host's cavity. Only the relatively bulky side-chains of TDZ and TFP were sufficiently locked to generate definite nuclear Overhauser effect (NOE) signals and to allow the definite assignment of an interaction with the secondary hydroxyl groups. For all phenothioazine ligands, binding poses in agreement with structural interpretation from NMR were obtained. They all reveal a bifurcated ammonium–hydroxyl interaction and an incorporation of the largely hydrophobic phenothiazine ring into the hydrophobic β-CD binding cavity (similar to Fig. 4).

The calculated Gibbs energy of binding of CPZ to β-CD (−6.7 kcal mol^−1^) is in good agreement with the experimental value of −5.4 kcal mol^−1^ (see Table S3, ESI†). In medicinal chemistry, introduction of a chlorine atom is frequently used to increase the lipophilicity of drug compounds.^17^ Promazine (PMZ) and chlorpromazine (CPZ) differ only in the replacement of a hydrogen atom by a chlorine, but chlorpromazine binds only slightly stronger than promazine (0.5 kcal mol^−1^ in experiment *vs.* 1.3 kcal mol^−1^ in calculations). This suggests that introducing the chlorine substituent is not significantly stabilizing ligand-receptor binding but may be beneficial for the drug's bioavailability. The calculated Gibbs energies of binding of all phenothiazine molecules at every level are given in Table S3 (ESI†).

Table 2 gives the analysis of errors of phenothiazine binding to β-CD. GFN2 is not able to give a binding of phenothiazine ligands to β-CD at all and the calculated Gibbs energy of binding is always positive (see Table S3 (ESI†)).

**Table 2:** Analysis of deviation of calculated (S)QM Gibbs energies of binding in kcal mol^−1^ for phenothiazine drug molecules to β-CD

	MSE^a^	SEM^b^	MAD^c^
GFN2	−18.4	2.3	18.4
Level 0	+4.8	1.5	4.8
Level 1	+2.1	1.9	2.4
Level 2	+2.4	0.4	2.4
Level 3	+0.4	0.7	0.7

a Mean signed error.

b Standard error of mean.

c Mean absolute deviation.

Semi-empirical methods, such as GFN2-*x*TB, have a poor performance in the description of ionic hydrogen bonds which are largely contributing to polarization.^29^ Such an underestimation of these strong hydrogen bonds can be assigned to the absence of polarization effects when using minimal valence basis sets in SQM.

As for the CB[8] receptor binding challenge, GFN2 generated structures are structurally very plausible but the deviation in energies from experiment is even larger. Calculations at level 0 and level 1 reduce the MSE and MAD significantly (see Table 2). Structural optimizations at level 2, however, do not lead to systematically lower errors for the SAMPL9 entries. High-level global hybrid *meta*-GGA XC functional DFT calculations generate very accurate Gibbs energies of binding with the targeted thermochemical accuracy (MSE + 0.4 kcal mol^−1^, MAD 0.7 kcal mol^−1^).

**Fig. 5:**
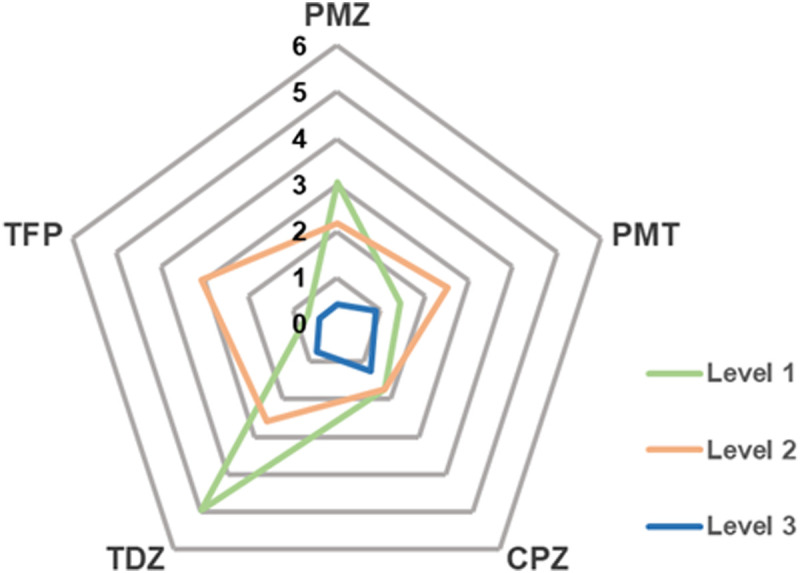
MAD from experiment in kcal mol^−1^ of calculated Gibbs energies of binding of phenothiazine drug molecules to β-CD.

Fig. 5 shows that the MAD from experimental data can systematically be reduced when going from level 1 to 3.

For β-CD phenothiazine binding, the best SAMPL9 submission was using an alchemical transfer model (ATM)^10^ with a proprietary ligand force field to give a MSE of −0.9 kcal mol^−1^ and a MAD of 1.6 kcal mol^−1^. In total, 64 individual alchemical Gibbs energy calculations and hundreds of nanoseconds of MD simulations needed to be performed obtain this accuracy. The combination of SQM exploration of the conformational space of ligands, receptors and complexes followed by refinement of electronic energies and solvation models is able to give results that are comparable to, or sometimes outperforming, the most accurate SAMPL9 submissions.

## Computational details

Ligands were manually positioned inside the receptor upon positioning. The initial complexes were first optimized using the GFN2-*x*TB 6.3.2 tight-binding method.^30^ Ensembles of structures were generated using CREST 2.12 and an ALPB^31^ continuum solvent representation for water. For thermostatistical corrections, GFN2-*x*TB single-point Hessian calculations (SPH^32^) and the modified rigid-rotor harmonic oscillator (mRRHO) was used.^33^ Solvent contributions were incorporated at various levels using ALPB, DCOSMO-RS^34^ and COSMO-RS^35,36^ solvation models. Throughout, Ahlrichs’ def2 basis sets and TURBOMOLE v7.5.1^26^ was used for all density functional theory (DFT) calculations.

To refine the Gibbs energies of the resulting CREs, commandline energetic sorting (CENSO v.1.2.0)^6^ was used. At ‘level 0’, electronic energies of all the conformers were recalculated using the low-cost semi-empirical GGA-type density functional with long range dispersion corrections B97-D3(0)^37,38^ with a small def2-SV(P)^39^ basis set and the analytical linearized Poisson–Boltzmann (ALPB) solvation model.^31^ All conformers within 4 kcal mol^−1^ from the minimum were further considered for the next level. ‘level 0’ only served to eliminate unrealistic high-energy conformers.

At ‘level 1’, the Gibbs energies of all remaining conformers were calculated from single point energy calculations using the composite electronic structure method of *meta*-GGA type r^2^SCAN-3c,^24,40^ a large purpose-built def2-mTZVPP basis set^41^ and D4 dispersion corrections.^42–44^ The Gibbs energy threshold for this level was set to be 3.5 kcal mol^−1^.

Structures from ‘level 1’ were then re-optimized at ‘level 2’ using the same r^2^SCAN-3c functional with def2-mTZVPP basis sets and D4 dispersion corrections. The effect of solvents were included using DCOSMO-RS^34^ during the geometry optimizations. This step gave DFT-optimized structures of the conformers and enabled the removal of further non-unique conformers. The thermal corrections were, again, re-calculated using SPH calculations with GFN2-*x*TB. The Gibbs solvation energies were estimated using COSMO-RS v19.0.4(R 5514)^35,45^ with TZVPD_FINE parameters for SAMPL8 and TZVPD parameters for SAMPL9 calculations. Finally, conformers within 2.5 kcal mol^−1^ energy threshold were retained.

A final electronic energy refinement (‘level 3’) was carried out using the computationally demanding PW6B95 (6-parameter functional based on Perdew–Wang-91 exchange and Becke-95 correlation) global-hybrid *meta*-GGA density functional with dispersion corrections.^38,46,47^ and a large def2-TZVPD basis set.^41^ The solvent effects were obtained from COSMO-RS.

•Level 0: SP B97-D3(0)/def2-SV(P) + *E*_solv_(GFN2-ALPB)

•Level 1: SP r^2^SCAN-3c(D4)/def2-mTZVPP + *E*_solv_(GFN-ALPB) + *G*_mRRHO_(GNF2-ALPB-SPH)

•Level 2: OPT r^2^SCAN-3c(D4)/def2-mTZVPP/DCOSMO-RS + *G*_solv_(COSMO-RS) + *G*_mRRHO_(GFN2-ALPB-SPH)

•Level 3: SP PW6B95-D3(BJ)^47^/def2-TZVPD + *G*_solv_(COSMO-RS) + *G*_mRRHO_(GFN2-ALPB-SPH)

Since calculations at ‘level 0’ are mere energies of binding, they cannot be directly compared with experiments and are not discussed in the main text.

## Conclusions

The Gibbs energy of binding of ligand-receptor complexes with thermochemical accuracy is a challenge to any computational approach. SAMPL challenges are an ideal opportunity to benchmark a large variety of different methods. The quantum chemical refinement of a moderate number of SQM poses in combination with an increasing level of description of electronic energies and (implicit) solvation is able to provide accurate Gibbs energies of binding of drug molecules to CB[8] and β-CD receptors. Accuracy of electronic energies, thermodynamic corrections and choice of solvent modelling are the critical ingredients here as they allow a systematic control and monitoring of their performance at various levels of refinement. The outlined SQM/QM approach is able to provide computational Gibbs energies of binding with comparable accuracy as experiment. System-specific force matching or force field parametrizations are not required. At every step in this systematic workflow, it also allows the control of accuracy of results and possible ranges of errors.

As an alternative to explicit molecular simulations, machine learning (ML) approaches are able to give Gibbs energies of binding. However, they require extensive network training on large datasets. For example, for the pillar[*n*]arene WP6,^48^ ML results trained on extensive experimental datasets were superior submissions to SAMPL9.^49^ For realistic or novel host–guest complexes,^50^ however, training data may sometimes be scarce. For example, the binding of phenothiazines to β-cyclodextrin was only recently investigated. Here, a ML framework to control the error of DFT calculations may be more appropriate.^51^

## Author contributions

FJ: investigation, formal analysis, writing – original draft, Data curation, methodology; MS: conceptualization, data

## Data availability

The data supporting this article have been included as part of the ESI.† Data for this article, including all structures, energies and ensembles at every level are available at zenodo at https://doi.org/10.5281/zenodo.10657702.

## Conflicts of interest

There are no conflicts to declare.

## Supplementary Material

CP-026-D4CP01529K-s001
