# Supplementary material for: Chemical accuracy for ligand-receptor binding Gibbs energies through multi-level SQM/QM calculations
Source: Phys Chem Chem Phys. 2024 Jul 19;26(31):21197–203. doi: 10.1039/d4cp01529k (PMC11305096; doi:10.1039/d4cp01529k)
Supplement: CP-026-D4CP01529K-s001 [file CP-026-D4CP01529K-s001.pdf]

Electronic Supplementary Material (ESI) for Physical Chemistry Chemical Physics.
This journal is © the Owner Societies 2024

Supplementary Information

Chemical accuracy for ligand-receptor binding Gibbs energies through multi-level SQM/QM calculations

Froze Jameel[a] and Matthias Stein[a,*]

[a]Molecular Simulations and Design Group, Max Planck Institute for Dynamics of Complex Technical Systems, Sandtorstrasse 1, 39106 Magdeburg

Corresponding author: Matthias Stein, matthias.stein@mpi-magdeburg.mpg.de

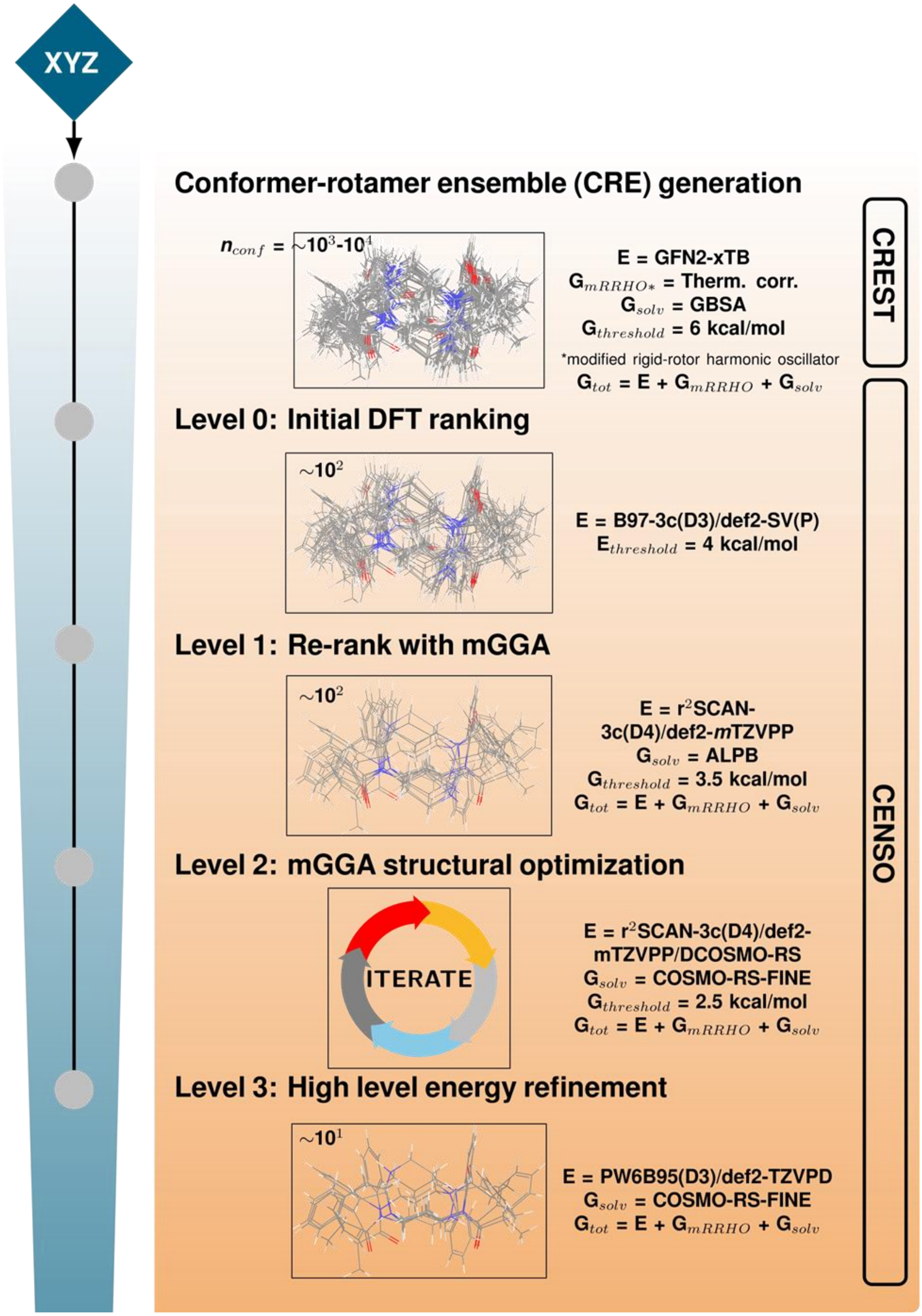

Figure S1. Schematic workflow used for conformational space exploration and sequential refinement of SAMPL8 and SAMPL9 ligand-receptor systems.

Details of calculated Gibbs energies of binding and their deviation from experiment

SAMPL8

Table S1. Experimental and calculated Gibbs energies of binding of 'drugs of abuse' molecules **G1** – **G9** to CB[8] in kcal/mol.

	**Exp.**	**GFN2**	**Level 0**	**Level 1**	**Level 2**	**Level 3**
**G1**	-7.05	-12.22	-26.56	-8.49	-6.53	-7.60
**G2**	-9.94	-11.14	-24.72	-7.46	-5.70	-8.00
**G3**	-11.6	-16.31	-28.75	-17.16	-8.43	-13.87
**G4**	-11.2	-16.13	-31.44	-17.34	-14.32	-15.86
**G5**	-12.3	-12.95	-26.07	-5.11	-10.84	-14.22
**G6**	-14.1	-11.3	-19.78	-9.46	-10.03	-14.52
**G7**	-7.93	-13.54	-31.69	-15.03	-5.08	-8.09
**G8**	-9.18	-7.9	-27.87	-5.37	-7.39	-5.61
**G9**	-10.2	-8.13	-28.17	-7.05	-9.40	-11.09

Table S2. Effect of the choice of solvent model for Level 2 calculations for SAMPL8 entries.

**Gibbs energy of binding to CB[8] in kcal/mol**		**r^{2}SCAN-3c/def2-mTZVPP**	
	**Exp.**	**COSMO-RS**	**ALPB**
**G1**	-7.05	-6.53	-9.10
**G2**	-9.94	-5.70	-9.18
**G3**	-11.6	-8.43	-18.81
**G4**	-11.2	-14.32	-15.95
**G5**	-12.3	-10.84	-11.67
**G6**	-14.1	-10.03	-11.61
**G7**	-7.93	-5.08	-16.17
**G8**	-9.18	-7.39	-8.41
**G9**	-10.2	-9.40	-11.13
**MAD**		2.45	3.09

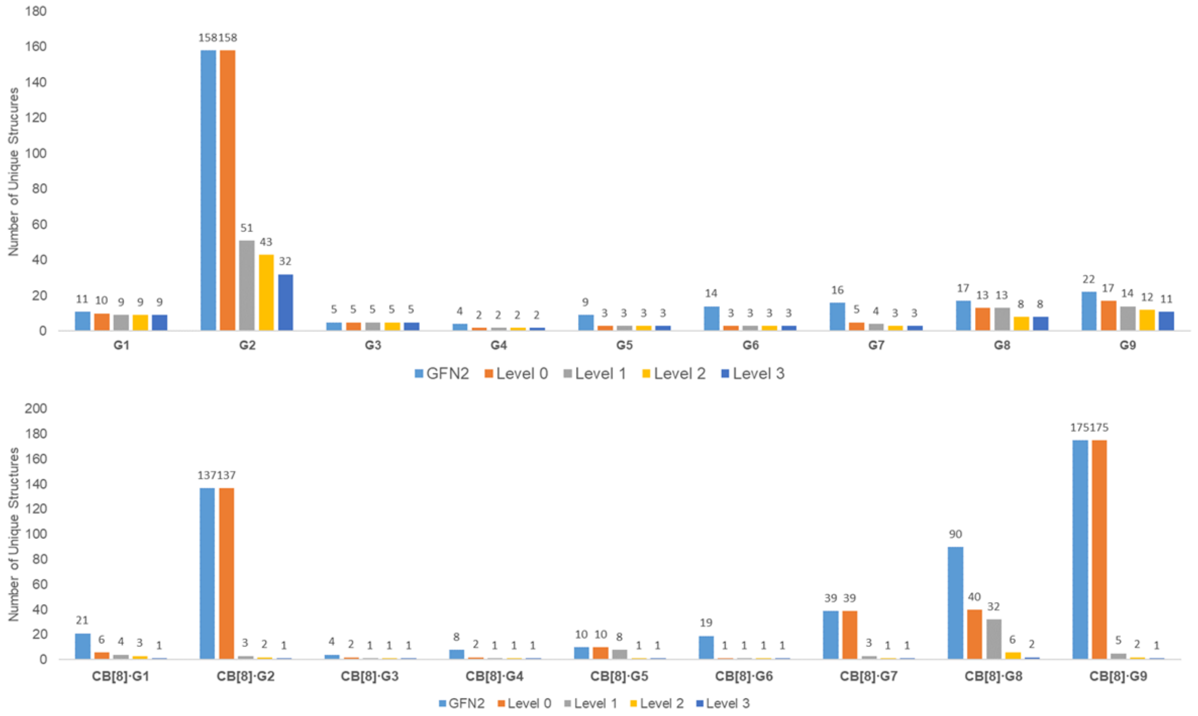

Figure S2. Number of unique structures of ligands **G1** – **G9** (top) and ligand-receptor complexes (bottom) for SAMPL8 at all levels of SQM and QM calculations.

Details of calculated Gibbs energies of binding and their deviation from experiment

SAMPL9

Table S3. Experimental and calculated Gibbs energies of binding of phenothiazine drug molecules to β-CD in kcal/mol.

	**Exp.**	**GFN2**	**Level 0**	**Level 1**	**Level 2**	**Level 3**
**Promazine (PMZ)**	-4.97	15.85	-7.51	-8.03	-7.14	-5.41
**Promethazine (PMT)**	-4.47	16.42	-9.88	-5.89	-7.00	-5.36
**Chlorpromazine (CPZ)**	-5.42	13.68	-12.17	-7.17	-7.15	-6.67
**Thioridazine (TDZ)**	-5.71	13.11	-11.22	-10.67	-8.30	-5.47
**Trifluoperazine (TFP)**	-5.06	8.98	-8.97	-4.41	-8.15	-5.47

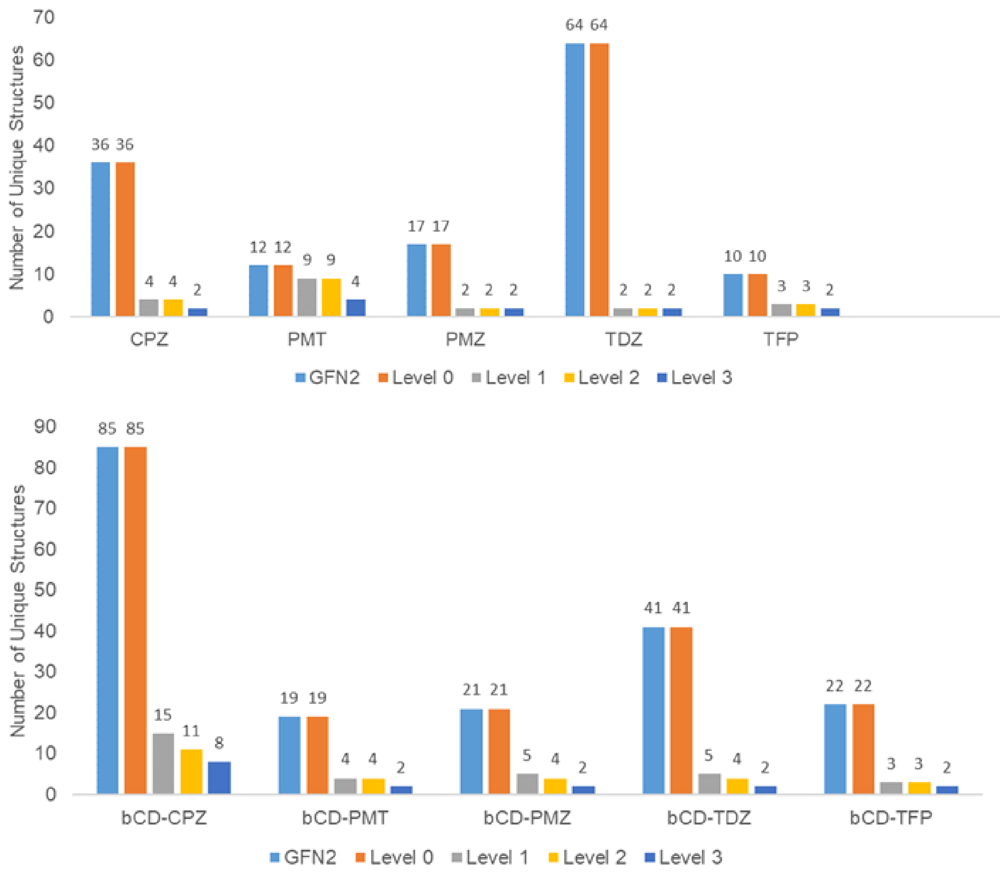

Figure S2. Number of unique structures of phenothiazine ligands (top) and ligand-receptor complexes (bottom) for SAMPL9 at all levels of SQM and QM calculations.

Table S4. Effect of choice of COSMO-RS level on Gibbs solvation energy for SAMPL8 entries in kcal/mol.

	**Exp.**	**COSMO-RS Model**	
		**FINE**	**Normal**
**G1**	-7.05	-7.60	-12.3
**G2**	-9.94	-8.00	-10.13
**G3**	-11.6	-13.87	-13.49
**G4**	-11.2	-15.86	-14.62
**G5**	-12.3	-14.22	-17.36
**G6**	-14.1	-14.52	-18.31

**G7**	-7.93	-8.09	-7.02
**G8**	-9.18	-5.61	-13.58
**G9**	-10.2	-11.09	-15.18
**MAD**		1.64	1.85

Table S5. Effect of choice of COSMO-RS level on Gibbs solvation energy for SAMPL9 entries.

	**Gsolv [Eh]**		**ΔGsolv [kcal/mol]**
	**COSMO-RS NORMAL**	**COSMO-RS FINE**	
bCD	-0.1459969	-0.1428208	-1.99
TDZ Ligand	-0.0590136	-0.0475236	-7.21
TDZ Complex	-0.1936377	-0.1932459	-0.25
CPZ Ligand	-0.0648921	-0.0527661	-7.61
CPZ Complex	-0.1994703	-0.2002071	0.46
PMT Ligand	-0.0630955	-0.049636	-8.45
PMT Complex	-0.2026583	-0.1989436	-2.33
PMZ Ligand	-0.0615527	-0.049128	-7.80
PMZ Complex	-0.2003445	-0.2014822	0.71
TFP Ligand	-0.0786545	-0.0613064	-10.89
TFF Complex	-0.2062826	not converged	
